# Modulatory Effect of Curcumin on Expression of Methyltransferase/Demethylase in Colon Cancer Cells: Impact on wt p53, mutp53 and c-Myc

**DOI:** 10.3390/molecules30153054

**Published:** 2025-07-22

**Authors:** Roberta Santarelli, Claudia Di Dio, Michele Di Crosta, Paola Currà, Roberta Gonnella, Mara Cirone

**Affiliations:** Department of Experimental Medicine, Sapienza University of Rome, 00161 Rome, Italy; didio.1753168@studenti.uniroma1.it (C.D.D.); michele.dicrosta@uniroma1.it (M.D.C.); roberta.gonnella@uniroma1.it (R.G.)

**Keywords:** colon cancer, c-Myc, p53, lysine methyltransferase, demethylases

## Abstract

Curcumin-mediated anti-cancer properties have been correlated with the inhibition of oncogenic molecules such as mutp53 and c-Myc. Their targeting is therapeutically significant, as p53, following point mutations, can acquire oncogenic functions, and c-Myc overexpression, due to translocations, point mutations, protein/protein interactions, or epigenetic modifications, plays a central role in cancer cell proliferation and metabolic reprogramming, particularly in colorectal cancer. In a previous study, we showed that curcumin strongly downregulated mutp53 while activating wtp53 and reduced the expression of methyltransferases such as EZH2, G9a, and MLL-1 in colon cancer cells. Based on this background, here we investigated whether the dysregulation of such methyltransferases could correlate with the effect observed on p53. We also explored whether these epigenetic changes could affect c-Myc expression in these cells. By Western blot analysis and RT-qPCR, we found that the downregulation of EZH2; G9a; and, to a lesser extent, KDM1, which was also reduced by curcumin, correlated with the decrease in mutp53 and that the reduction of EZH2 and KDM1 correlated with the activation of wtp53. Regarding c-Myc, we unveiled the occurrence of a positive feedback loop between it and MLL-1, which was inhibited by curcumin, independently of the p53 status. In conclusion, this study provides new insights into the therapeutic potential of curcumin, which involves its properties to act as an epigenetic modulator and target key molecules in colon cancer cells.

## 1. Introduction

Curcumin, a polyphenolic compound derived from the rhizome of *Curcuma longa*, traditionally used in Ayurvedic and Chinese medicine, has garnered significant attention for its broad spectrum of pharmacological properties, which include anti-inflammatory and anti-cancer activities [1]. Regarding cancer, curcumin has been reported to modulate multiple cellular signaling pathways, resulting in the reduction of cell proliferation, angiogenesis, and metastasis [2].

One of the most compelling aspects of curcumin’s action is its ability to influence epigenetics, being able to alter DNA methylation, histone methylation and acetylation, and the expression of non-coding RNAs [3]. Therefore, curcumin emerges as a promising compound for cancer prevention and treatment, particularly in malignancies such as colorectal cancer, in which epigenetic abnormalities play a pivotal role [4]. Colorectal cancer remains one of the most common and lethal malignancies worldwide, representing a major public health burden [5]. Carcinogenesis of the colon is a multistep process driven by the accumulation of both genetic and epigenetic alterations that progressively transform normal colonic epithelium into carcinoma. Indeed, in addition to key genetic mutations affecting *APC*, *KRAS*, and *TP53*, it is emerging that epigenetic dysregulation contributes to colorectal tumorigenesis [6]. It includes aberrant DNA methylation, histone modifications, and altered expression of non-coding RNAs, resulting in heritable changes in gene expression without modifying the underlying DNA sequence [7]. Epigenetic changes can lead to the silencing of tumor suppressor genes; lead to the activation of oncogenes; and disrupt cellular processes such as differentiation, apoptosis, and DNA repair [8]. In colon cancer in particular, epigenetic silencing of *CDKN2A* has been shown to contribute to cell cycle dysregulation. Moreover, promoter hypermethylation of *MLH1*, a DNA mismatch repair gene, represents a hallmark of microsatellite instability-high (MSI-H) colon cancer [9].

In addition, key oncogenes and tumor suppressor genes, including wtp53, mutant p53 proteins, and c-Myc, may undergo epigenetic changes, which amplify their oncogenic potential.

The reversible nature of epigenetic changes offers a unique therapeutic opportunity, with epigenetic drugs and natural compounds, such as curcumin, being actively investigated for their potential to restore normal gene expression profiles of oncogenes and tumor suppressors. In a recent study, we have shown that the curcumin-induced cytotoxic effect against colon cancer cells correlated with the downregulation of methyltransferases such as enhancer of Zeste 2 Polycomb repressive complex 2 Subunit (EZH2), G9a, and mixed lineage leukemia protein-1 (MLL-1). Moreover, curcumin induced the activation of wtp53 and the reduction of mutp53 in these cells [10].

Here, we correlated the methyltransferase reduction by curcumin with the activation of wtp53 and the downregulation of mutp53 and found that the changes in these methyltransferases and demethylase such as KDM1 affected both wt and mutp53. Another important finding of this study is that changes in these epigenetic enzymes correlated with the downregulated expression of c-Myc, another molecule targeted by curcumin in wtp53, mutp53, and also p53^-/-^ colon cancer cells.

## 2. Results

### 2.1. Methyltransferase/Demethylase Modulation Correlates with wtp53 Stabilization and mutp53 Downregulation in Colon Cancer Cells Treated by Curcumin

**Figure 1:**
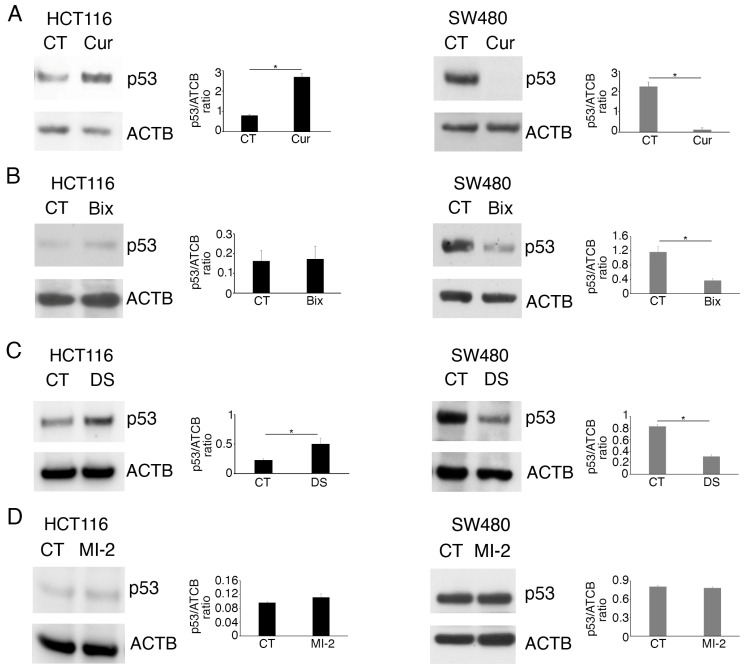
Inhibition of EZH2 and G9a methyltransferases contributes to wtp53 stabilization and mutp53 downregulation in colon cancer cells. Western blot analysis showing the level of wtp53 and mutp53 in HCT116 and SW480 colon cell lines cultured for 24 h in the presence of (**A**) Cur 25 µM; (**B**) G9a inhibitor Bix-01294 (Bix) 3 µM; (**C**) EZH2 inhibitor DS-3201 (DS) 5 µM; and (**D**) Menin inhibitor MI-2, 2.5 µM. Untreated cells were used as the control (CT) and DMSO as a vehicle. Actin B (ACTB) was used as the loading control. The histograms represent the mean plus SD of the densitometric analysis of the ratio of p53/ACTB of three different experiments. * indicates a *p*-value < 0.05.

In a previous study, we have shown that curcumin induced a cytotoxic effect in colon cancer cells, which correlated with the downregulation of Histone Lysine Methyltransferases (KMTs) such as EZH2, G9a, and MLL-1. Moreover, we showed that curcumin downregulated mutp53 and activated wtp53 in SW480 and HCT116 cells, respectively [10]. In the present study, we confirmed that curcumin alters wtp53 and mutp53 expression in these cells (Figure 1A) and investigated whether these effects were correlated with the changes in the expression of KMTs. To this end, HCT116 and SW480 cells were treated with the specific inhibitors of G9a, of EZH2, or of the Menin partner of MLL-1, namely Bix-01294 (Bix), Valemetostat/DS-3201 (DS), or MI-2, respectively. We found that DS upregulated wtp53 in HCT116, and that, similarly to Bix, it reduced the expression level of mutp53 in SW480 cells (Figure 1B,C). The Menin inhibitor MI-2 instead slightly affected wtp53 and mutp53 in colon cancer cells (Figure 1D).

**Figure 2:**
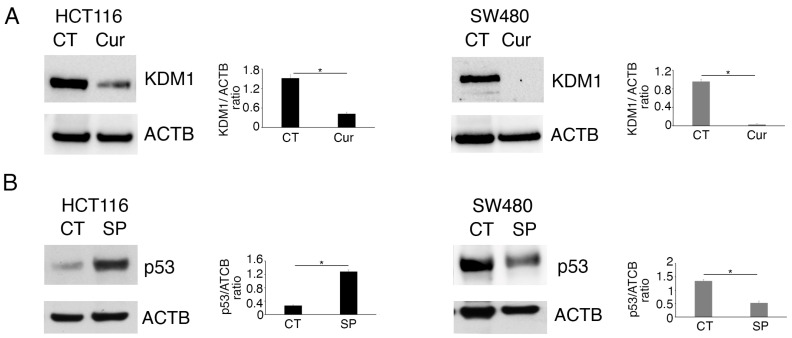
Curcumin reduction of KDM1 correlates with the wtp53 and mutp53 regulation in HCT116 and SW480 cell lines. Western blotting to evaluate (**A**) KDM1 expression in HCT116 and SW480 cell lines cultured in the presence of Cur 25 µM for 24 h and (**B**) the level of wtp53 and mutp53 in HCT116 and SW480 cells grown in the presence of KDM1 inhibitor SP2509 (SP) 1.5 µM. Untreated cells were used as control (CT) while Actin B (ACTB) as loading control. The histograms represent the mean plus SD of the densitometric analysis of the ratios of KDM1/ACTB and p53/ACTB of three different experiments. * indicates a *p*-value < 0.05.

We then explored if curcumin could dysregulate the expression level of one of the most important demethylases, namely, Lysine-Specific Histone Demethylase 1 (KMD1/LSD1), which mainly targets the H3K4 lysine methylation and is known to demethylate p53, as well [11]. We found that KDM1 was reduced by curcumin both in the HCT116 and the SW480 cell lines (Figure 2A) and that its inhibition by SP2509 (SP) strongly upregulated wtp53 and, to a lesser extent, reduced mutp53 (Figure 2B).

**Figure 3:**
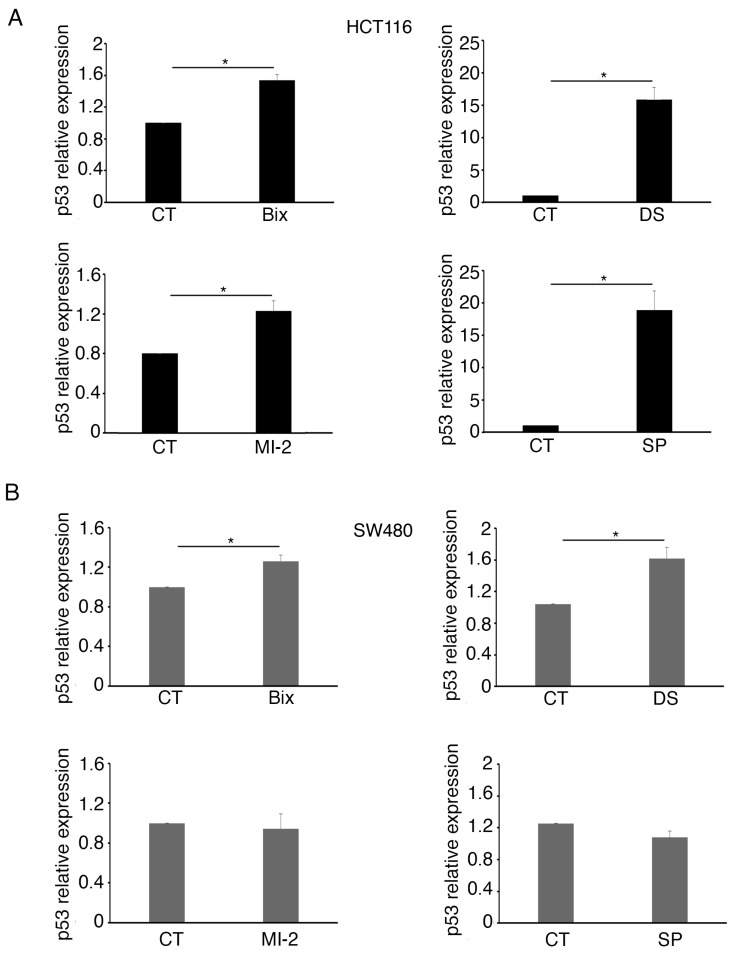
G9a, EZH2, MLL-1, and KDM1 regulate wtp53 mRNA expression while slightly modulating mutp53 mRNA. p53 mRNA levels were quantified by RT-qPCR in (**A**) HCT116 and (**B**) SW480 cell lines cultured in the presence of Bix 3 µM or DS 5 µM or MI-2 2.5 µM or SP 1,5µM for 24 h. Beta-actin was used as the reference gene. Untreated cells were used as the control (CT). The histograms represent the mean plus SD of three different experiments. * indicates a *p*-value < 0.05.

As wtp53 and mutp53 were regulated by curcumin also at the mRNA level, we next evaluated the impact of G9a, EZH2, MLL-1, and KDM1 inhibition on wt and mutp53 mRNAs. As shown in Figure 3A,B, and recapitulated in Supplementary Table S1, the effect induced by Bix, DS, MI-2, and SP on mRNAs mirrored those observed on wtp53 protein in HCT116. Instead, SP, Bix, and DS, although they reduced the protein, they slightly affected mutp53 mRNA, suggesting that this protein was mainly regulated at the post-transcriptional level by these epigenetic enzymes. Accordingly, in our previous study, we showed that mutp53 underwent lysosomal degradation following curcumin treatment [10]. Overall, these results suggest that curcumin-induced changes in KMT/KDM expression may contribute to the effects observed on wtp53 and mutp53, acting in the first case at the transcriptional level while acting mainly at the post-transcriptional level on mutp53.

### 2.2. Curcumin Reduces the Expression of c-Myc in mutp53, wtp53, and p53^-/-^ Colon Cancer Cells

**Figure 4:**
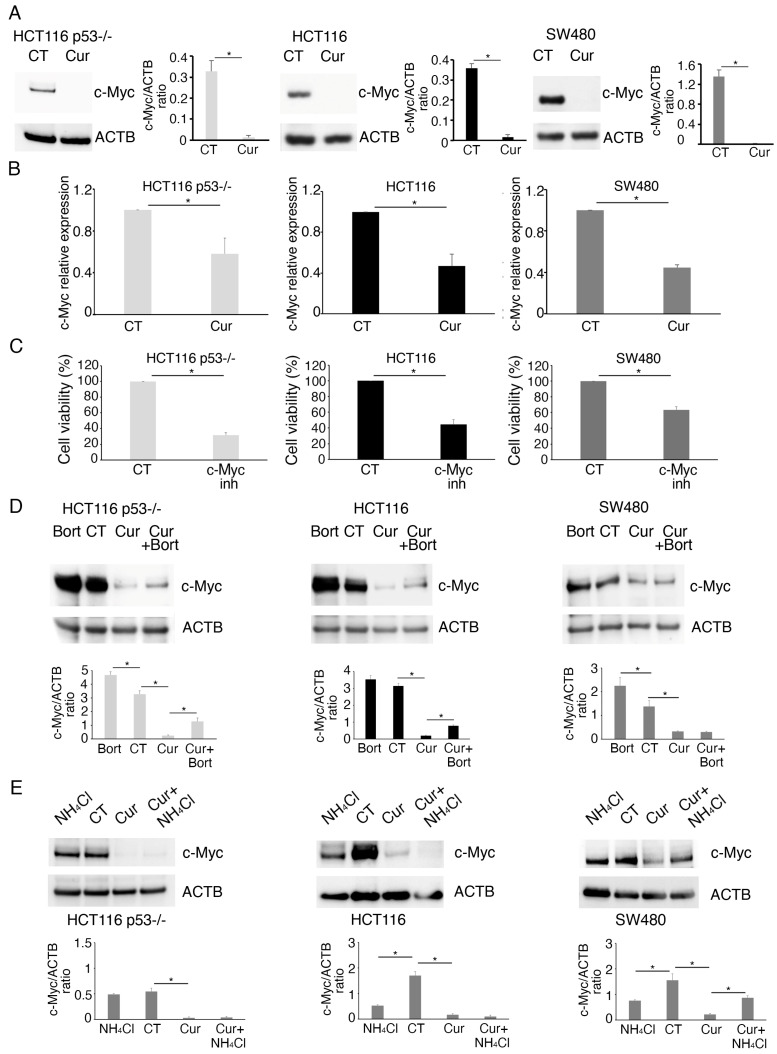
The expression of c-Myc is reduced by curcumin in p53^-/-^, wtp53, and mutp53 colon cancer cell lines. (**A**) Western blot analysis to assess the level of c-Myc in HCT116 p53^-/-^ and wtp53, as well as in SW480, cell lines cultured in the presence of Cur 25 µM for 24 h. Untreated cells were used as control (CT). Histograms represent the mean plus SD of the densitometric analysis of the ratio of c-Myc/ACTB of three different experiments. * indicates a *p*-value < 0.05. (**B**) RT-qPCR to evaluate the c-Myc mRNA level in HCT116 p53^-/-^ and wtp53, as well as in SW480, cell lines cultured in the presence of Cur 25 µM for 24 h. Untreated cells were used as the control (CT). GAPDH was used as the reference gene. The histograms represent the mean plus SD of three different experiments. * indicates a *p*-value < 0.05. (**C**) Trypan blue assay on HCT116 p53^-/-^ and wtp53, as well as on SW480, cell lines grown in the presence of c-Myc inhibitor 65 μM for 24 h. Untreated cells were used as the control (CT). The histograms represent the mean plus SD of three different experiments. * indicates a *p*-value < 0.05. Western blotting analysis showing the level of c-Myc (**D**) in HCT116 p53^-/-^ and wtp53, as well as in SW480, cell lines cultured in the presence of Cur 25 µM for 24 h and incubated with a proteasome inhibitor, Bortezomib (Bort, 20 nM), during the last 6 h, (**E**) or with NH_4_Cl 40 µM during the last 6 h. Untreated cells were used as control (CT) while Actin B (ACTB) as loading control. Histograms represent the mean plus SD of the densitometric analysis of the ratio of c-Myc/ACTB of three different experiments. * indicates a *p*-value < 0.05.

As we have previously shown that curcumin was cytotoxic also against HCT116 p53^-/-^ cells, we searched for molecular targets other than p53 possibly affected by curcumin. For this purpose, we investigated the impact of curcumin on c-Myc, as this is one of the molecules most involved in driving colon cancer cell proliferation [12], and as curcumin has been reported to interfere with c-Myc activity [13]. The results obtained indicate that curcumin reduced c-Myc expression both at the protein (Figure 4A) and the mRNA level (Figure 4B), an effect contributing to the cytotoxicity of curcumin, as demonstrated by the reduction of cell proliferation following c-Myc inhibition (Figure 4C). As c-Myc protein was more strongly reduced compared to its mRNA, we then investigated if curcumin could also promote c-Myc protein degradation. We found that proteasome inhibition by bortezomib partially rescued c-Myc in wtp53 and p53^-/-^ HCT116 treated by curcumin (Figure 4D), suggesting a partial degradation of this protein though this route. Differently from HCT116, proteasome inhibition did not influence c-Myc in SW480 cells (Figure 4D), while its expression was rescued by inhibiting lysosomal degradation by NH_4_Cl (Figure 4E). On the other hand, NH_4_Cl-mediated lysosomal inhibition did not rescue the expression of c-Myc in HCT116 p53^-/-^, HCT116 (Figure 4E), and wtp53 RKO cells (Supplementary Figure S1). Accordingly, we have previously shown that lysosomes contributed to c-Myc degradation in Akata cells, a Burkitt Lymphoma cell line in which this protein is carried in a mutated form [14], and, interestingly, it has been reported that c-Myc is mutated also in SW480 cells [15]. This suggests that the route involved in c-Myc degradation might be influenced by the presence of mutations, although other mechanisms, such as different post-translational modifications occurring in wt and mutp53 cells, could be responsible for a different c-Myc destiny.

### 2.3. MLL1 and KDM1 Sustain c-Myc Expression in Colon Cancer Cells

**Figure 5:**
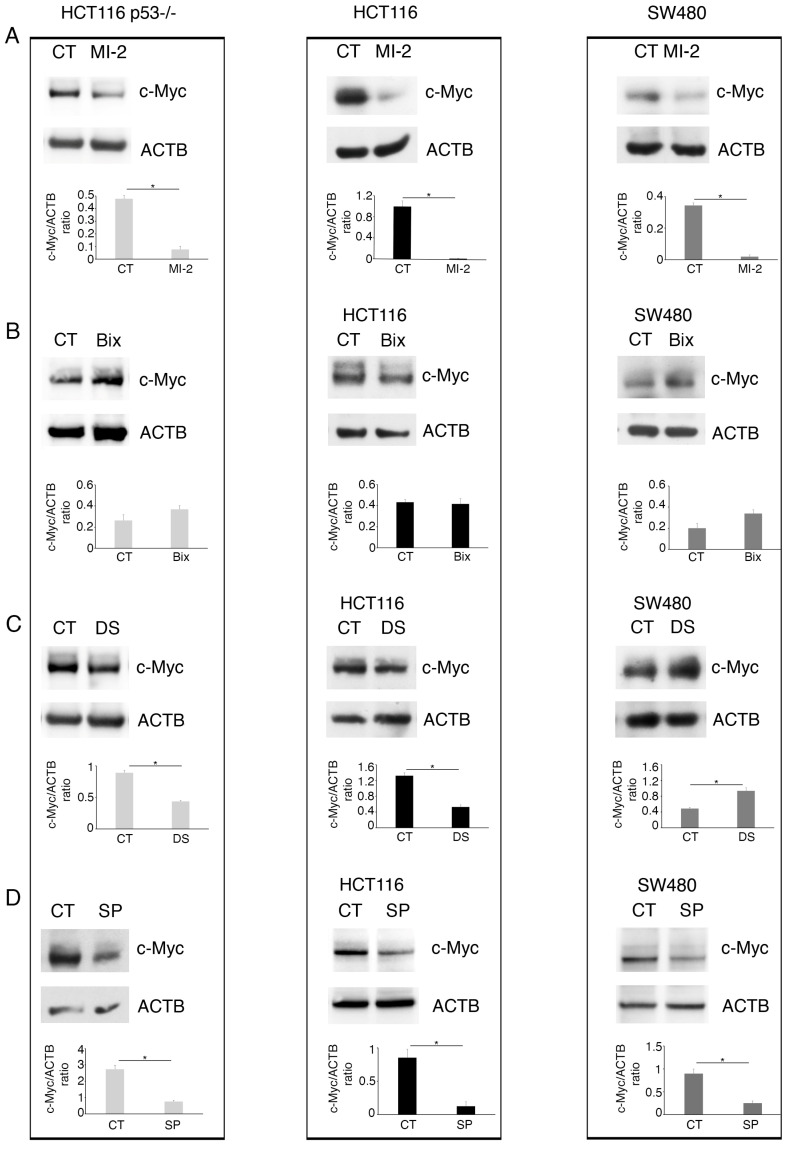
The reduction of the c-Myc protein level correlates with the downregulation of MLL-1 in mutp53, wtp53, and p53^-/-^ colon cancer cell lines. c-Myc protein expression in HCT116 p53^-/-^ and wtp53, as well as in SW480, cell lines cultured for 24 h in the presence of (**A**) MI-2 2.5 µM, (**B**) Bix 3 µM, (**C**) DS 5 µM, and (**D**) SP 1.5 µM, assessed by Western blotting. Untreated cells were used as control (CT) while Actin B (ACTB) as loading control. Histograms represent the mean plus SD of the densitometric analysis of the ratio of c-Myc/ACTB of three different experiments. * indicates a *p*-value < 0.05.

**Figure 6:**
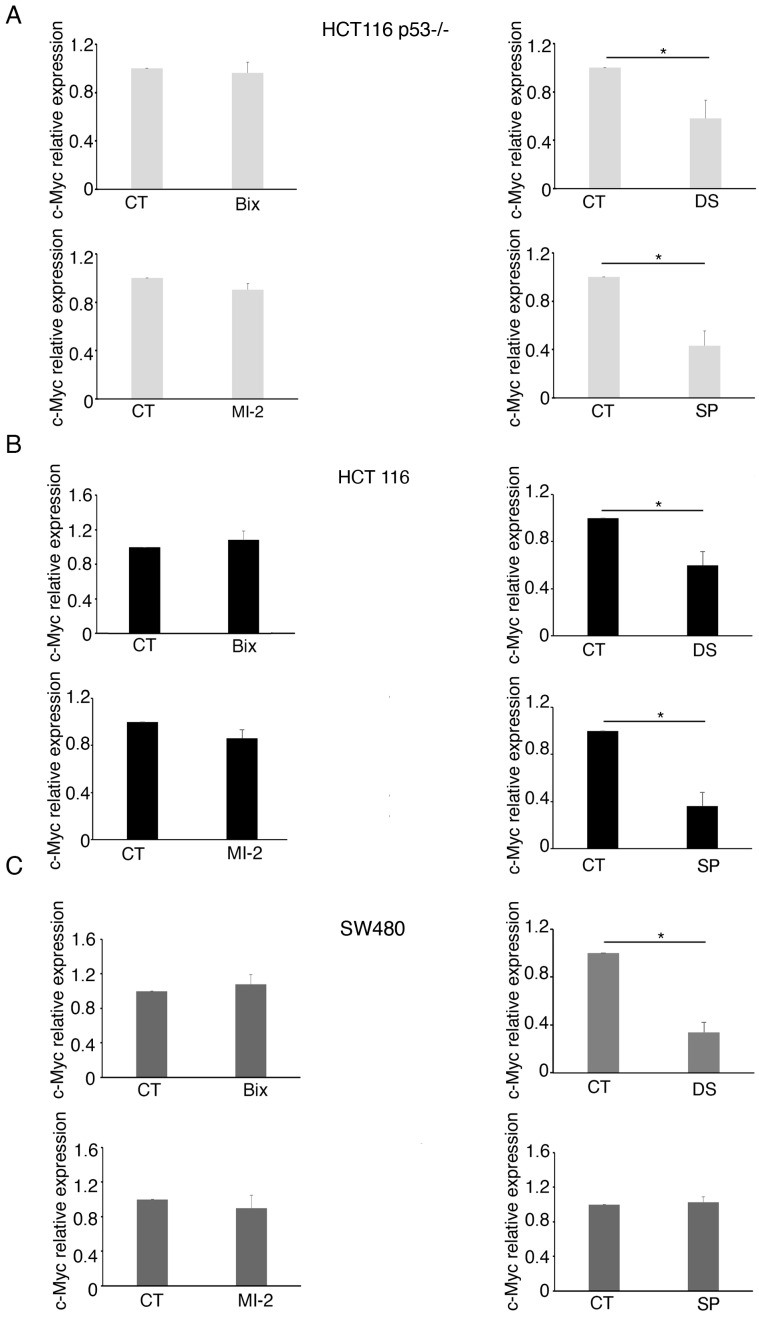
The modulation of c-Myc mRNA expression by KMT/KDM differs in p53^-/-^ and wtp53 colon cancer cell lines compared to SW480. c-Myc mRNA levels evaluated by RT-qPCR in (**A**) HCT116 p53^-/-^, (**B**) HCT116, and (**C**) SW480 cell lines cultured in the presence of Bix 3 µM or DS 5 µM or MI-2 2.5 µM or SP 1.5 µM for 24 h. GAPDH was used as the reference gene. Untreated cells were used as control (CT). The histograms represent the mean plus SD of three different experiments. * indicates a *p*-value < 0.05.

Next, we evaluated if G9a, EZH2, MLL-1, and KDM1, the enzymes downregulated by curcumin, could contribute to c-Myc reduction. At this aim, HCT116 and SW480 cells were treated by Bix, DS, MI-2, or SP and analyzed for the c-Myc expression level. As shown in Figure 5A, c-Myc was reduced by MI-2 in all the cell lines, independently of the p53 status, while EZH2 inhibitor downregulated it in HCT116 (Figure 5B), suggesting the wt and mutp53 could differently influence the activity of EZH2. The inhibition of KDM1 by SP was able to downregulate c-Myc in both cell lines, although to a lesser extent compared to MI-2 (Figure 5D). As shown in Figure 6A,B and recapitulated in Supplementary Table S2, C, RT-qPCR evidenced that DS also reduced c-Myc mRNA, particularly in SW480, while in HCT116, p53^-/-^, and wt, SP was more effective in inducing this effect. Finally, MI-2 slightly affected c-Myc mRNA in colon cancer cells, suggesting that it was reducing c-Myc mainly acting at the post-transcriptional level.

### 2.4. c-Myc Alters Methyltransferase/Demethylase Expression in Colon Cancer Cells

**Figure 7:**
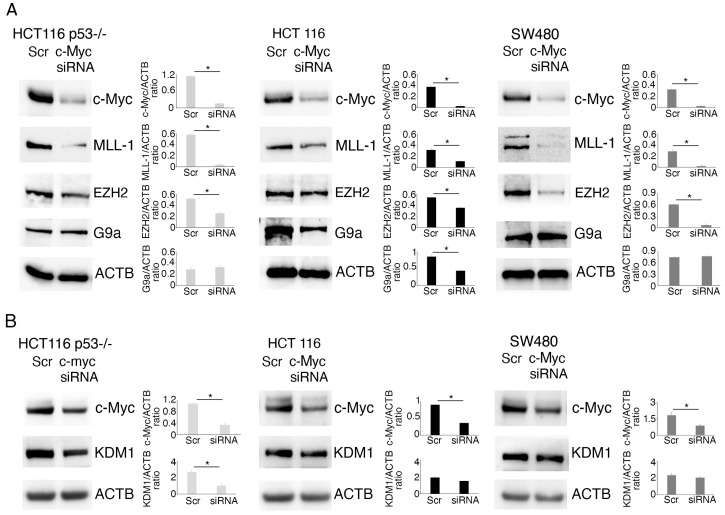
Methyltransferase/demethylase expression in colon cancer cells is regulated by c-Myc. Western blotting to assess the level of (**A**) c-Myc, MLL-1, EZH2, and G9a and (**B**) c-Myc and KDM1 in HCT116 p53^-/-^ and wtp53, as well as in SW480, cell lines transfected with a *c-Myc* siRNA or with a scrambled (Scr) siRNA-A as control for 48 h. Untreated cells were used as control (CT) while Actin B (ACTB) as loading control. The histograms represent the mean plus SD of the densitometric analysis of the ratio of c-Myc/ACTB, MLL-1/ACTB, EZH2/ACTB, G9a/ACTB, and KDM1/ACTB of three different experiments. * indicates a *p*-value < 0.05.

We then explored if c-Myc could influence the expression level of KMT/KDM in colon cancer cells. To address this question, we silenced c-Myc and found that MLL-1 was strongly reduced in silenced cells compared to the scramble-treated control, independently of the p53 status (Figure 7A). This suggests the occurrence of a positive feedback loop between c-Myc and MLL-1 in colon cancer cells, which was interrupted by curcumin. We also found that *c-Myc* silencing downregulated EZH2 mainly in mutp53 SW480, while it reduced G9a in wtp53 HCT116 cells, and that the KDM1 expression level was slightly affected (Figure 7A,B). Given the importance of KMTs such as MLL-1, EZH2, and G9a [16,17,18] in carcinogenesis, their upregulation by c-Myc may contribute to its oncogenic potential, and thus their inhibition may be exploited to counteract c-Myc tumorigenesis. Similar results on MLL-1, the epigenetic enzyme more strongly downregulated by *c-Myc* silencing, were obtained by pharmacological inhibition of c-Myc (Supplementary Figure S2).

## 3. Discussion

Epigenetic manipulation holds great promise for the treatment of cancer, especially colon cancer, which is driven also by epigenetic abnormalities [4]. Colon cancer can be classified into hereditary, sporadic, and colitis-associated [19], and, for example, the high microsatellite instability (MSI) in sporadic colon cancers often relies on epigenetic modifications (e.g., methylation of the promoter of *MLH1*, *MSH2*, etc.) [20]. Curcumin is considered an epigenetic modulator, and, in a previous study, we confirmed this property by demonstrating that this polyphenol induces profound changes in lysine methylation in colon cancer cells, activating wtp53 in HCT116 cells while reducing mutp53 in SW480 cells [10]. In this study, we explored the correlation between curcumin-mediated downregulation of KMTs such as EZH2, G9a, and MLL-1 and the effects induced on p53. We also evaluated the influence of these enzymes modulated by curcumin on c-Myc expression in these cells. We found that EZH2 inhibition modulated, although in opposite ways, the expression level of wtp53 and mutp53; the G9a inhibitor downregulated mutp53; and the inhibition of KDM1, a KDM reduced by curcumin, increased wtp53 expression, while it slightly influenced mutp53. Although CHIP experiments will be required to elucidate if the specific lysine methylation mediated by these KTM/KDM may occur at the promoters of the proteins, the results of this study suggest that *wtp53* was influenced at the transcriptional level by these epigenetic enzymes, while *mutp53* was regulated mainly at the post-transcriptional level, being that the mRNA encoding it was slightly affected by KMT/KDM inhibitors. Of note and in line with these results, in our previous study, we reported that curcumin promoted the lysosomal degradation of mutp53 [10]. Therefore, it is plausible to hypothesize that mutp53 reduction by curcumin may contribute to increased protein degradation, which may be promoted by post-translational modifications consequent to the reduction of KMTs such as EZH2 and G9a. The relationship between wtp53 or mutp53 and EZH2 has been previously explored in other cell types [21,22,23], while regarding G9a, its role has been evaluated with respect to wtp53 [24]. Previous studies have reported that also KDM1 regulates wtp53 [11,25]; but, as with G9a, the impact of this KDM on mutp53 requires further investigations. In agreement with the results of the above reported studies [11,25], we found that the inhibition of KDM1 by SP2509 led to the upregulation of wtp53, which suggests that the increased expression of wtp53 by curcumin may involve the reduction of KDM1.

**Figure 8:**
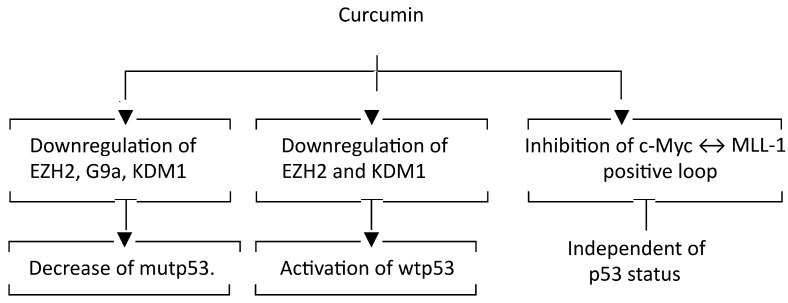
Diagram showing how changes in methyltransferases/demethylase induced by curcumin affect mutant p53, wtP53 and c-Myc.

Another important finding of the present study is that curcumin regulates *c-Myc* expression, unveiling that this natural compound may interfere with the criminal alliance occurring between c-Myc and MLL-1. Accordingly, Menin, a protein essential for MLL-1 function, has previously been shown to enhance c-Myc-mediated transcription promoting cancer progression [26], and c-Myc, in turn, has been reported to be able to hijack H3K4 methylation, mediated by MLL-1, to promote tumorigenesis [27]. However, the inhibition of MLL-1 did not affect c-Myc mRNA, suggesting that this KMT was mainly acting at the post-transcriptional level, an aspect that will be worth investigating in future studies.

c-Myc downregulation correlated with EZH2 inhibition in SW480 and with G9a inhibition in HCT116 cells, suggesting that the presence of wtp53 or mutp53 could influence the activity of KMTs in colon cancer cells. *c-Myc* can be considered one of the main triggers of colon cancer, and its overexpression may be related to epigenetic or genetic abnormalities. Interestingly, we found that mutations seem to render c-Myc more susceptible to lysosomal degradation following curcumin treatment, as we observed that NH_4_Cl partially restored its expression level in SW480 cells harboring *c-Myc* in a mutated form. A similar result was obtained in a previous study, in which Akata BL cells harboring the *c-Myc* mutation were treated with quercetin [14]. In addition to linking methylation with the expression of oncogenic molecules, here we show that c-Myc in turn regulates epigenetics in colon cancer cells, being able to sustain not only the expression of MLL-1 but also that of EZH2. In conclusion, this study demonstrates that curcumin-induced modulation of wtp53 and oncogenic molecules, such as mutp53 and c-Myc, can correlate with changes in KMT and KDM expression, as represented in the following diagram (Figure 8).

This could help find new therapeutic opportunities where natural compounds, but also specific epigenetic drugs, could be used to improve the treatment of aggressive tumors such as colon cancer, whose onset and progression depend, as mentioned above, on both genetic abnormalities and epigenetic changes. This is particularly important in the case of colorectal cancer since it continues to be the second most deadly cancer worldwide [5]. Understanding the interplay between epigenetic modifications and oncogenic molecules in aggressive tumors may enable the advancement of diagnostic biomarkers and the development of targeted epigenetic therapies, which could ultimately improve patient outcomes.

## 4. Materials and Methods

### 4.1. Cell Cultures, Reagents, and Treatments

The human colorectal carcinoma cell lines HCT116 p53^-/-^, wild-type (wt) p53 HCT116 (HCT116), and RKO, as well as mutant (mut) p53 SW480 (p53R273H and p53P309S), were cultured in Dulbecco’s modified Eagle medium (DMEM Sigma Aldrich, St. Louis, MO, USA), supplemented with 10% fetal bovine serum (FBS; Sigma Aldrich, St. Louis, MO, USA), L-glutamine (40 mM) (Invitrogen, Waltham, MA, USA), streptomycin (100 µg/mL) (Corning, New York, NY, USA), and penicillin (100 U/mL) (Corning, New York, NY, USA), at 37 °C in a 5% CO_2_ incubator.

For the different experiments, 3 × 10^5^ cells were plated/well in 6-well plates and, 24 h after seeding, were cultured for a further 24 h in the presence of:

- Curcumin (Cur), (C1386, Sigma Aldrich, St. Louis, MO, USA), 25 µM;
- G9a inhibitor Bix-01294 (Bix), (HY-10587, MedChemExpress Monmouth Junction, NJ 08852, USA), 3 µM;
- EZH2 inhibitor DS-3201 (DS, Valemetostat, Selleckchem, Cologne, Germany),
- 5 µM;
- Menin inhibitor MI-2 (HY-15222, MedChemExpress Monmouth Junction, NJ 08852, USA), 2.5 µM;
- KDM1 inhibitor SP2503 (SP) (HY-12635, MedChemExpress Monmouth Junction, NJ 08852, USA), 1.5 µM;
- c-Myc inhibitor, (475956, Sigma Aldrich, St. Louis, MO, USA), 65 µM.

HCT116 p53^-/-^, HCT116, and SW480 were seeded at a density of 3 × 10^5^ cells/well in 6-well plates, cultured in the presence of Cur 25 µM for 24 h and then, for proteasome inhibition, cells were incubated with bortezomib (20 nM) for the final 6 h prior to harvest. Phagosome–lysosome fusion inhibition was instead achieved by adding NH_4_Cl 40 µM to SW480 and RKO cells during the final 6 h of curcumin treatment.

DMSO (Sigma Aldrich, St. Louis, MO, USA) was used as a vehicle, and the compounds were dissolved to ensure a final DMSO concentration of ≤0.1%. Untreated cells were used as the control (CT) in all the experiments performed.

### 4.2. c-Myc Silencing

To silence *c-Myc*, HCT116 p53^-/-^, HCT116, and SW480 cells were plated in 6-well plates (2 × 10^5^ cells/well) and transfected with either *c-Myc*-specific siRNA (si c-Myc; Santa Cruz Biotechnology Inc., Dallas, TX, USA, sc-29226) or Control siRNA-A (scrambled, Scr; Santa Cruz Biotechnology Inc., Dallas, TX, USA, sc-37007), using INTERFERin^®^ reagent (Polyplus-transfection, Illkirch-Graffenstaden, France) according to the supplier’s instructions. After 48 h, cell viability was determined, and cells were collected for Western blotting.

### 4.3. Cell Viability

To evaluate cell viability following treatment with the c-Myc inhibitor, HCT116 p53^-/-^, HCT116, and SW480 cells were subjected to the Trypan Blue exclusion assay (Sigma Aldrich, 72571). Viable cells were identified as unstained and counted using a Neubauer chamber and bright-field microscopy.

### 4.4. Western Blotting

HCT116 p53^-/-^, HCT116, and SW480 cells were harvested by centrifugation and lysed using RIPA buffer (150 mM NaCl, 1% NP-40, 50 mM Tris-HCl pH 8.0, 0.5% sodium deoxycholate, and 0.1% SDS), supplemented with protease and phosphatase inhibitors.

Next, protein samples (10 µg per lane) were separated on pre-cast Bolt™ 4–12% Bis-Tris Plus polyacrylamide gels (Invitrogen, Waltham, MA, USA) and transferred onto nitrocellulose membranes (Bio-Rad, Hercules, CA, USA).

The membranes were briefly stained with Ponceau S solution (SERVA Electrophoresis GmbH, Heidelberg, Germany, 33,427.01) to verify protein transfer, then cut into different slices and blocked for 30 min in PBS containing 0.1% Tween-20 and 2% BSA.

Then, each membrane slice was incubated, for 1 h at room temperature or overnight at 4°, with a specific primary antibody diluted in the blocking solution. Following three washes in PBS-Tween-20 (0.1%), membranes were incubated with HRP-conjugated secondary antibodies, also diluted in the blocking solution, for 30 min at room temperature. After additional washes in PBS Tween-20 0.1%, a Western Bright ECL chemiluminescence kit (Advansta, Menio Park, CA, USA) was used for protein detection.

### 4.5. Antibodies

For Western blotting analysis, the following primary antibodies were used: mouse monoclonal anti-p53 (DO-1) (1:100) (Santa Cruz Biotechnology, Dallas, TX, USA, Sc-126); mouse monoclonal anti-β-Actin (AC-74) (1:10,000) (Sigma-Aldrich, A5441); mouse monoclonal anti-G9a (C-9) (1:100) (Santa Cruz Biotechnology, Dallas, TX, USA, Sc-515726); rabbit polyclonal anti-EZH2 (1:1000) (Proteintech, Rosemont, IL 60018, USA, #21800-1-AP); rabbit monoclonal anti-Anti-MLL1 (1:1000) (Bethyl Laboratories, Montgomery, TX, USA, #A300-086A); rabbit polyclonal anti-KDM1 (1:8000) (Proteintech, Rosemont, IL 60018, USA, #20813-1-AP); rabbit polyclonal anti-c-Myc (1:500) (Proteintech, Rosemont, IL 60018, USA, #10828-1-AP). Goat anti-rabbit IgG-HRP (1:40,000) (Bethyl Laboratories, #A120-101P) and Goat anti-mouse IgG-HRP (1:20,000) (Bethyl Laboratories, #A90-116P) were used as secondary antibodies.

### 4.6. Densitometric Analysis

Protein bands were quantified by densitometric analysis using ImageJ software (1.54p version, NIH, Bethesda, MD, USA; https://imagej.net).

### 4.7. RNA Extraction, Reverse Transcription, and Quantitative Real-Time Polymerase Chain Reaction (qRT-PCR)

Total RNA was isolated from untreated cells and from cells treated for 24 h with Cur 25 µM or with the above-mentioned methyltransferase and demethylase inhibitors. RNA extraction was carried out using TRIzol™ Reagent (Life Technologies Corporation, Carlsbad, CA, USA), according to the manufacturer’s protocol. To eliminate potential genomic DNA contamination, samples were treated with RNase-free DNase I (1 U/mL; Norgen Biotek, Thorold, ON, Canada) for 10 min at room temperature. Next, reverse transcription was performed using the High-Capacity cDNA Reverse Transcription Kit (Applied Biosystems, Waltham, MA, USA), and quantitative real-time PCR was carried out using the SensiFast SYBR No-ROX Kit (Bioline, Memphis, TN, USA). Gene expression levels were normalized to β-actin as the reference gene.

The primers used were as follows:

TP53 Fw 5′–GTGTGGAGTATTTGGATGAC–3′;

TP53 Rev 5′–GTCAGAGCCAACCTCAG–3′;

ACTIN Fw 5′–TCATGAAGTGTGACGTGGACATC–3′;

ACTIN Rev 5′–CAGGAGGAGCAATGATCTTGATCT–3′

The TaqMan gene expression assay was used to evaluate the c-Myc mRNA expression level (Applied Biosystem HS00153408), and GAPDH was used as the reference gene (Applied Biosystem, HS99999905-m1).

The 2^−∆∆Ct^ method was utilized to normalize gene transcription data.

### 4.8. Statistical Analysis

Data are presented as mean ± standard deviation (SD) from three independent experiments. Statistical comparisons were performed using the Student’s *t*-test. Differences were considered statistically significant at *p* < 0.05 and are marked with an asterisk (*) in the figures. Non-significant differences (*p* ≥ 0.05) are not indicated.

## Data Availability

The datasets generated and analyzed during the current study are available from the corresponding author upon reasonable request.

## Supplementary Materials

The following supporting information can be downloaded at: https://www.mdpi.com/article/10.3390/molecules30153054/s1, Figure S1. Western blotting analysis showing the level of c-Myc in wtp53 RKO cell line cultured in presence of Cur 25 μM for 24 h and incubated with NH4Cl 40 μM during the last 6 h. Figure S2. MLL-1 protein expression in HCT116 p53^-/-^ and wtp53 as well as in SW480 cell lines cultured for 24 h in the presence of c-Myc inhibitor 65 μM, evaluated by Western blotting. Table S1. Impact of G9a, EZH2, Menin and KDM1 inhibitors on wtp53 and mutp53. Table S2. Impact of G9a, EZH2, Menin and KDM1 inhibitors on c-Myc in p53^-/-^, wtp53 and mutp53 cell lines.

## Abbreviations

The following abbreviations are used in this manuscript:

MSI-H	microsatellite instability-high
EZH2	enhancer of Zeste 2 Polycomb repressive complex 2 Subunit
MLL-1	mixed lineage leukemia protein-1
KMD1	Lysine-Specific Histone Demethylase 1 (KMD1/LSD1)
KMTs	Histone Lysine Methyltransferases
