# Supplementary material for: Modulatory Effect of Curcumin on Expression of Methyltransferase/Demethylase in Colon Cancer Cells: Impact on wt p53, mutp53 and c-Myc
Source: Molecules. 2025 Jul 22;30(15):3054. doi: 10.3390/molecules30153054 (PMC12348775; doi:10.3390/molecules30153054)
Supplement: Supplementary file 1 [file molecules-30-03054-s001.zip › molecules-3724950-supplementary.pdf]

Supplementary Figure S1. Western blotting analysis showing the level of c-Myc in wtp53 RKO cell line cultured in presence of Cur 25 μM for 24 h and incubated with NH4Cl 40 μM during the last 6 h. Untreated cells were used as control (CT) while Actin B (ACTB) as loading control. Histograms represent the mean plus SD of the densitometric analysis of the ratio of c-Myc/ACTB of three different experiments. * indicates p-value < 0.05.

Supplementary Figure S2. MLL-1 protein expression in HCT116 p53 -/- and wtp53 as well as in SW480 cell lines cultured for 24 h in the presence of c-Myc inhibitor 65 μM, evaluated by Western blotting. Untreated cells were used as control (CT) while Actin B (ACTB) as loading control.

Supplementary Table S1. Impact of G9a, EZH2, Menin and KDM1 inhibitors on wtp53 and mutp53

Supplementary Table S2. Impact of G9a, EZH2, Menin and KDM1 inhibitors on c-Myc in p53 -/-, wtp53 and mutp53 cell lines

**Treatment**	**wtp53**	**mutp53**
G9a Inhibitor (Bix-01294)	↑	↑
EZH2 inhibitor (DS-3201)	↑↑↑	↑
Menin inhibitor (MI-2)	↑	=
KDM1 inhibitor (SP2509)	↑↑↑↑	=

Supplementary table S1

Treatment	C-MYC	C-MYC	C-MYC
G9a Inhibitor (Bix-01294)	=	=	=
EZH2 inhibitor (DS-3201)	↓	↓	↓↓↓
Menin inhibitor (MI-2)	=	=	=
KDM1 inhibitor (SP2509)	↓↓	↓↓↓	=
	**HCT116 p53-/-**	**HCT116**	**SW480**

Supplementary table S 2

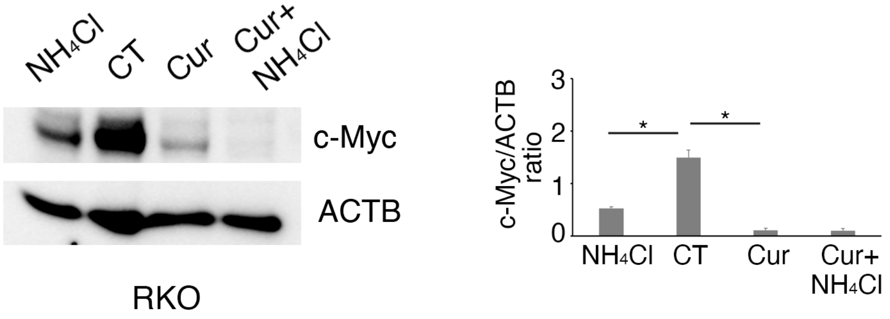
NH4Cl
CT
Cur
Cur+ NH4Cl
c-Myc
ACTB
RKO
c-Myc/ACTB ratio
3
2
1
0
*
*
NH4Cl
CT
Cur
Cur+ NH4Cl

Figure S1

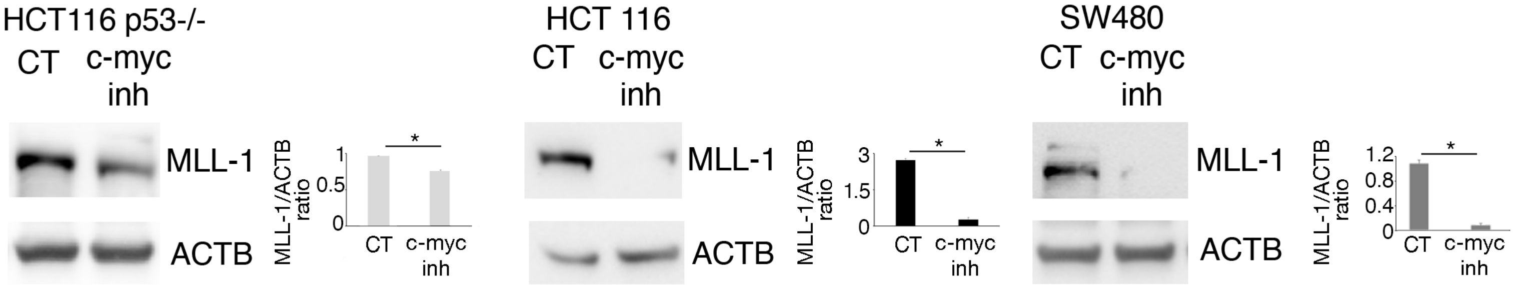
HCT116 p53-/-
CT
c-myc inh
MLL-1
ACTB
MLL-1/ACTB ratio
1
0.5
0
CT
c-myc inh
*
HCT 116
CT
c-myc inh
MLL-1
ACTB
MLL-1/ACTB ratio
3
1.5
0
CT
c-myc inh
*
SW480
CT
c-myc inh
MLL-1
ACTB
MLL-1/ACTB ratio
1.2
0.8
0.4
0
CT
c-myc inh
*

Figure S2
